# Structure and Magnetic Properties of ErFe_x_Mn_12−x_ (7.0 ≤ x ≤ 9.0, Δx = 0.2)

**DOI:** 10.3390/nano12091586

**Published:** 2022-05-07

**Authors:** Penglin Gao, Yuanhua Xia, Jian Gong, Xin Ju

**Affiliations:** 1 School of Mathematics and Physics, University of Science and Technology Beijing, Beijing 100083, China; gaoyingluo@163.com; 2 Key Laboratory of Neutron Physics and Institute of Nuclear Physics and Chemistry, China Academy of Engineering Physics, Mianyang 621900, China; xiayuanhua2009@126.com (Y.X.); gongjian@hotmail.com (J.G.)

**Keywords:** neutron diffraction, exchange-bias, magnetocaloric effect

## Abstract

The magnetic interactions of iron-rich manganese-based ThMn_12_ type rare earth metal intermetallic compounds are extremely complex. The antiferromagnetic structure sublattice and the ferromagnetic structure sublattice had coexisted and competed with each other. Previous works are focus on studying magnetic properties of RFe_x_Mn_12−x_ (x = 0–9.0, Δx = 0.2). In this work, we obtained a detailed magnetic phase diagram for iron-rich ErFe_x_Mn_12−x_ series alloy samples with a fine composition increment (Δx = 0.2), and studied the exchange bias effect and magneto-caloric effect of samples. ErFe_x_Mn_12−x_ series (x = 7.0–9.0, Δx = 0.2) alloy samples were synthesized by arc melting, and the pure ThMn_12_-type phase structure was confirmed by X-ray diffraction (XRD). The neutron diffraction test was used to confirm the Mn atom preferentially occupying the 8i position and to quantify the Mn. The magnetic properties of the materials were characterized by a comprehensive physical property measurement system (PPMS). Accurate magnetic phase diagrams of the samples in the composition range 7.0–9.0 were obtained. Along with temperature decrease, the samples experienced paramagnetic, ferromagnetic changes for samples with x < 7.4 and x > 8.4, and paramagnetic, antiferromagnetic and ferromagnetic or paramagnetic, ferromagnetic and antiferromagnetic changes for samples with 7.4 ≤ x ≤ 8.2. The tunable exchange bias effect was observed for sample with 7.4 ≤ x ≤ 8.2, which resulting from competing magnetic interacting among ferromagnetic and antiferromagnetic sublattices. The maximum magnetic entropy change in an ErFe_9.0_Mn_3.0_ specimen reached 1.92 J/kg/K around room temperature when the magnetic field change was 5 T. This study increases our understanding of exchange bias effects and allows us to better control them.

## 1. Introduction

Manganese (Mn) is the only 3d-series element that forms a stable ThMn_12_-type structure with rare earth elements [1,2], and it is mainly ferromagnetic and antiferromagnetic [3]. However, the pure ThMn_12_-type rare earth iron compound RFe_12_ does not exist. In the early 1980s, Yang et al. [4] found that a stable ternary rare earth iron intermetallic compound R(Fe_x_Mn_1−x_)_12_ could be formed by substitution, thus setting off a surge of research into iron-rich ThMn_12_-type compounds [5]. Subsequent studies have found that a number of tertiary elements can stabilize the ThMn_12_ phase; their molecular formulas can be written as RFe_x_M_12−x_ or R(Fe,M)_12_, where R is a rare earth element and M = Mn, V, Cr, Mo, W, Ti, Si, Al, Nb or Ga [6,7,8,9].

In the RMn_12_ alloy, the strong antiferromagnetic interaction between manganese atoms prohibits interaction between rare earth atoms and manganese atoms, so the RMn_12_ alloy has two magnetic ordering temperatures: R-R ferromagnetic ordering temperature, and Mn-Mn antiferromagnetic ordering temperature [10]. Iron (Fe) can replace Mn in large quantities (up to 75%) without changing the crystal structure [11]. Researchers [12,13,14,15,16,17,18,19,20,21,22] have investigated the structure and magnetic transitions of RFe_x_Mn_12−x_-series materials (x = 0–9.0, Δx = 1) using neutron diffraction, magnetic measurements and electrical measurements and have found that magnetic interaction in the alloy is extremely complex. As the proportion of Fe increases, the material undergoes an antiferromagnetic → antiferromagnetic + ferromagnetic → ferromagnetic transition. Among the iron-rich RFe_x_Mn_12−x_-series (x = 6.0–9.0) samples, only materials with integer values of x have been studied. This composition range includes the magnetic transition stage in which antiferromagnetism and ferromagnetism coexist in the material and plane anisotropy and axis anisotropy compete with each other. Therefore, it is necessary to prepare iron-rich RFe_x_Mn_12−x_-series (x = 6.0–9.0) alloy samples with a finer composition change to obtain more detailed and complete magnetic phase diagrams, and thus be able to develop new aspects of applications for the material. We first studied YFe_x_Mn_12−x_-series (x = 6.0–9.0) samples to obtain more complete magnetic phase diagrams for the materials and observed very large exchange bias effects and zero field cooling (ZFC) exchange bias effects in the samples [23]. After the discovery of exchange bias effect in Co/CoO nanoparticles, investigations have been mainly focused on a large number of heterogeneous structures such as magnetic bilayers, core-shell nanoparticles, and ferromagnetic nanoparticles embedded in antiferromagnetic matrix compounds [24,25,26]. So, it is necessary to further study exchange bias for the bulk metallic materials with exchange interactions occurring among the bulk sublattice. Firstly, we study how the magnetic atoms affect the EB effect in ThMn_12_-type compounds. The second-order Stevens factor *α_J_* for Er atoms is >0, but the second-order crystal field coefficient (A20) of the rare earth sublattice in the ThMn_12_ structure is negative, so magnetocrystalline anisotropy tends to the easy axis. We prepared ErFe_x_Mn_12−x_-series (7.0 ≤ x ≤ 9.0, Δx = 0.2) alloy specimens have been prepared by arc melting to enable us to investigate the structure and magnetism of the alloy.

## 2. Experimental Methods

ErFe_x_Mn_12−x_-series (7.0 ≤ x ≤ 9.0, Δx = 0.2) alloys were prepared by arc melting. The raw material was melted 4–5 times in an argon gas atmosphere according to the stoichiometric ratio to produce the alloy ingot; 5% more rare earth and 13% more Mn were added to compensate for volatilization in the melting process. A smaller current of 150 A was applied twice for melting, followed by a 200 A current once or twice to control the against excessive Mn volatilization. Specimens from the master alloy ingots were placed in sealed quartz tubes filled with argon and cooled down to room temperature after heat treatment at 1173 K for 2 days.

Phase purity was confirmed by a Cu target X-ray powder diffractometer (PANalytical, Almelo, The Netherlands) at room temperature. The high-resolution neutron diffraction spectrometer (*λ* = 0.18846 nm) of Mianyang Research Reactor (CMRR, Mianyang, China) was used to analyze the crystal structure, in particularly for the positions of Mn atoms. Powdered alloy was bonded into a small cylinder with epoxy resin or the alloy ingot was shattered, so that we could select a small piece of regular shape for magnetic measurement. The ZFC and field cooling (FC) thermomagnetic curves (*M−T* curves) of the samples were recorded, and the magnetic hysteresis loops (*M−H* loops) of the samples under different FC and temperature conditions were measured by the comprehensive physical property measurement system (PPMS, Quantum Design (San Diego, CA, USA)).

## 3. Experimental Results and Analysis

A phase of the ThMn_12_-type structure was formed in the ErFe_x_Mn_12−x_-series (7.0 ≤ x ≤ 9.0) ingots, and some samples contained a small quantity of the Er(Fe, Mn)_2_ phase. Heterogeneous Er_2_(Fe, Mn)_17_ and Er(Fe, Mn)_2_ phases are formed in ErFe_x_Mn_12−x_-series (7.0 ≤ x ≤ 9.0) alloys after heat treatment above 1273 K, which differentiates them from YFe_x_Mn_12−x_-series (7.0 ≤ x ≤9.0) alloys. Long duration high-temperature heat treatment is therefore not suitable for this series of materials; 1173 K heat treatment for 48 h will produce homogeneous alloy samples with good crystal shapes.

**Figure 1:**
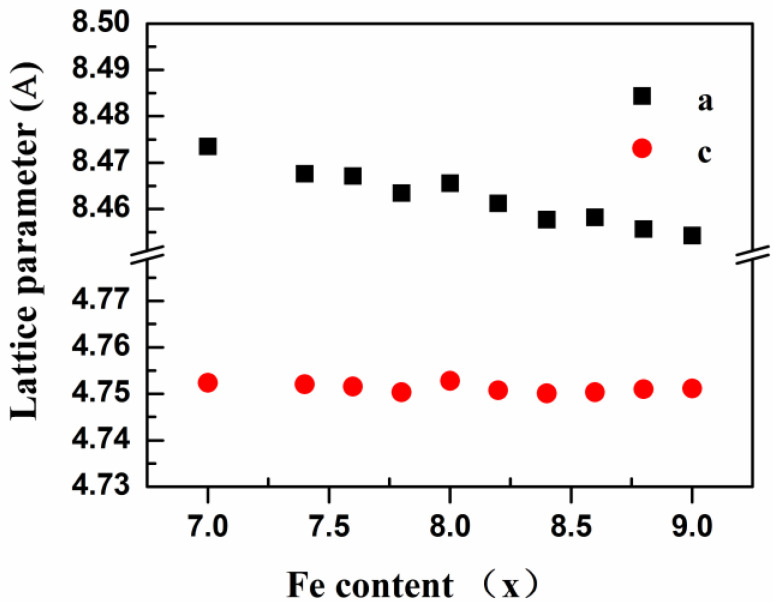
Variation of lattice constants *a* and *c* with Fe content of ErFe_x_Mn_12−x_ (7.0 ≤ x ≤ 9.0) series alloys after heat treatment.

The X-ray diffraction (XRD) spectra of the samples were examined before and after heat treatment. FullProf software [27] was used to refine the structure of the samples after heat treatment, and the relationship between the lattice constant and the composition of the samples was determined, as shown in Figure 1. With the increasing proportion of Fe, the lattice constant *a* decreased linearly and *c* remained unchanged.

**Figure 2:**
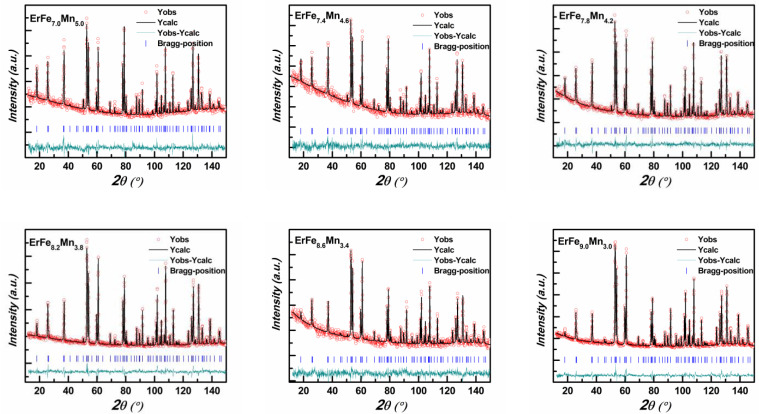
Refined neutron diffraction pattern of ErFe_x_Mn_12−x_ (7.0 ≤ x ≤ 9.0) series alloys (where red dots are experimental data, black curves are theoretical simulations, blue vertical bars are Bragg diffraction peak positions and the bottom green solid line is the difference curve).

The complete neutron diffraction spectra of some heat-treated samples were examined at room temperature, and the structure was refined using FullProf. The fitting spectrum is shown in Figure 2, and the crystal structure parameters are shown in Table 1. The samples formed a pure ThMn_12_-type phase of space group I4/mmm (139), with rare earth Er atoms occupying the 2a position and Fe and Mn occupying three other unequal positions (8i, 8j, and 8f). Since the coherent neutron scattering lengths of Mn atoms (b_Mn_ = −0.39) and Fe atoms (b_Fe_ = 0.95) are significantly different, the relative proportions of Fe and Mn in the alloy samples can be obtained by fitting neutron diffraction data; the results are shown in Table 1. The Mn atom occupies the 8i position preferentially. The trend of change in the proportion of Mn in the materials was similar to that of the initial materials, although the proportion of Mn was slightly higher, which indicated that the proportion of compensated Mn in the initial materials was relatively high. The lattice constant *a* decreased as the proportion of Fe decreased, while the lattice constant *c* remained basically unchanged. This is because the Mn atom preferentially occupies the 8i position, and 8i–8i lies in the plane ab. Changes in the proportion of Mn therefore greatly influences the lattice constants *a* and *b* but has little effect on the lattice constant *c*.

**Figure 3:**
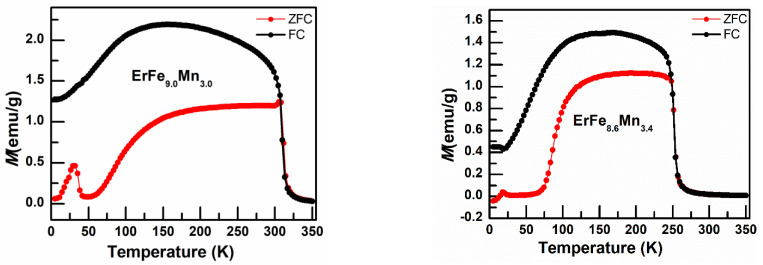

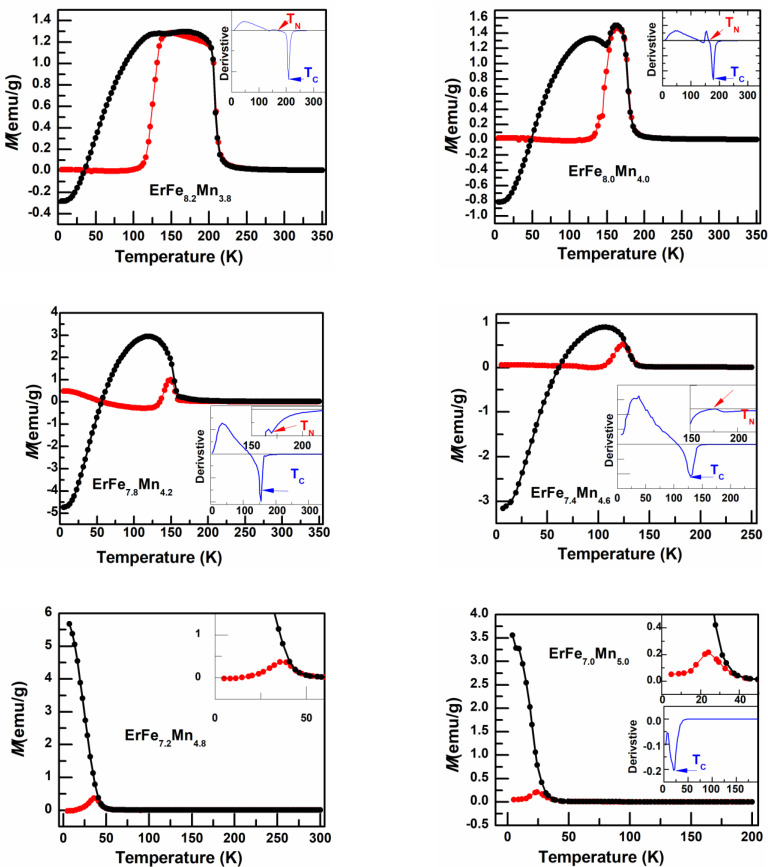
*M*−*T* curves for ErFe_x_Mn_12−x_ (7.0 ≤ x ≤ 9.0) series alloys under zero field cooling (ZFC) and field cooling (FC) conditions, H = 50 Oe. (The inset shows the *M*−*T* curves under FC after differentiation.)

Figure 3 shows the thermomagnetic curves of ErFe_x_Mn_12−x_-series (7.0 ≤ x ≤ 9.0) alloy samples in an external magnetic field of 50 Oe. *T_C_* represents the Curie temperature, *T_N_* is the Néel temperature, *T_C_* and *T_N_* is obtained by differentiating the *M*−*T* curves under FC. *T_f_* is the temperature corresponding to the bifurcation point in the ZFC and FC magnetization curves. As can be seen from the figure, the ZFC and FC *M−T* curves of the samples both clearly bifurcated as the temperature decreased. *T_f_* was slightly lower than the paramagnetic–ferromagnetic transition temperature of the samples due to the coexistence of Er-Er and Fe-Fe ferromagnetic exchanges interactions. Er-Fe, Er-Mn, Fe-Mn and Mn-Mn antiferromagnetic exchanges interactions, all interactions compete with each other, leading to spin frustration in the samples at low temperatures. For samples with x > 7.2, the FC *M−T* curves initially increased to the maximum value and then decreased gradually as the temperature decreased. The curve steepened, and both the speed and amplitude of bending increased as the proportion of Fe decreased; it reached the maximum for x = 7.8 and then began to decrease and disappeared for x = 7.2. The magnetization curves for x > 7.2 samples were typical of ferrimagnetism magnetization curves. This was because light rare earth lattices and metal lattices are ferromagnetically arranged and heavy rare earth lattices and metal lattices are antiferromagnetically arranged in rare earth intermetallic compounds with a ThMn_12_-type structure. Er is a heavy rare earth atom, so the samples had a ferrimagnetic structure in which the lattice magnetic moments of rare earth and transition metals were inversely arranged. As the temperature decreased, the magnetic moments of rare earth in the lattice increased rapidly and magnetic moments of transition metals increased slowly; the total magnetic moments of the samples initially increased to the maximum value and then decreased rapidly, and even showed a negative magnetic susceptibility. The x = 7.2 and x = 7.0 samples behave like pure ferro- or ferrimagnetic samples where high coercivity has developed already close to *T_C_*. This causes the maximum in the ZFC curves very close to *T_C_*.

In the YFe_x_Mn_12−x_-series (6.0 ≤ x ≤ 8.8) samples, as the proportion of Fe decreased, the *T_C_* of the alloy rapidly decreased and the *T_N_* slowly increased; the antiferromagnetic exchange magnetic ordering temperature of Mn-Mn was observed [23]. After rare earth Er atoms with magnetic moments replaced Y atoms without magnetic moments, the antiferromagnetic order of Mn-Mn was suppressed; the obvious antiferromagnetic order of Mn-Mn was only observed in the samples with the Fe proportion 7.4 ≤ x ≤ 8.2. The magnetic ordering temperature is shown in Table 2. Similar to YFe_x_Mn_12−x_, the ferromagnetic transition temperature of the alloy materials decreased rapidly as the proportion of Fe decreased.

**Figure 4:**
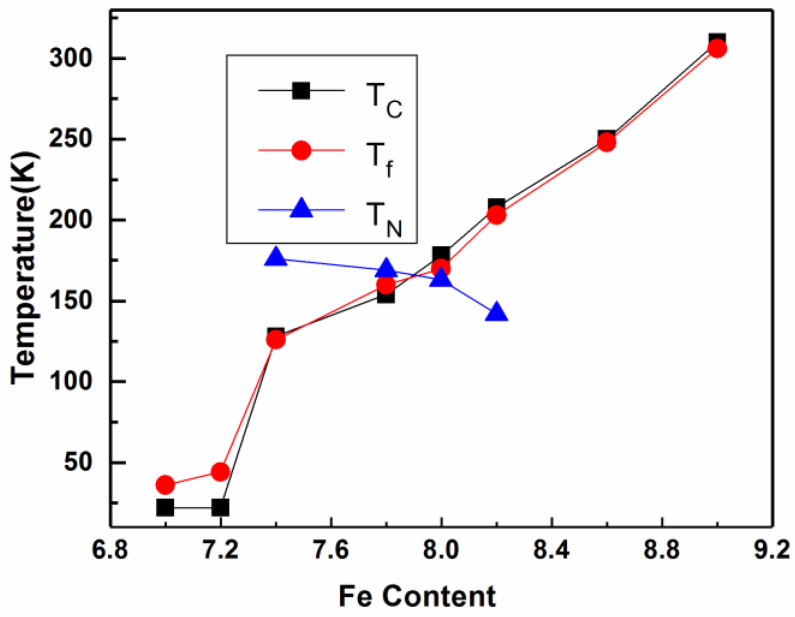
The magnetic phase diagram of ErFe_x_Mn_12−x_ (7.0 ≤ x ≤ 9.0) series alloys.

**Figure 5:**
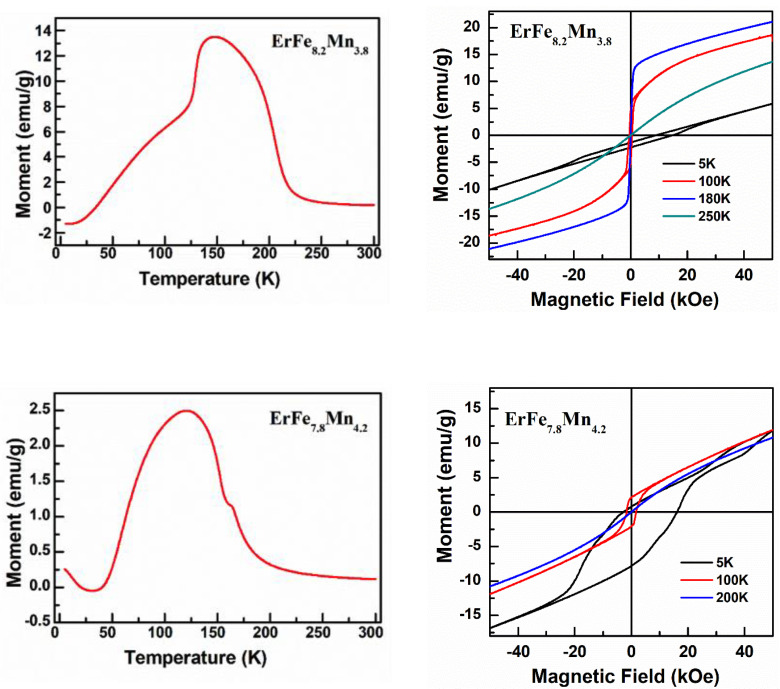

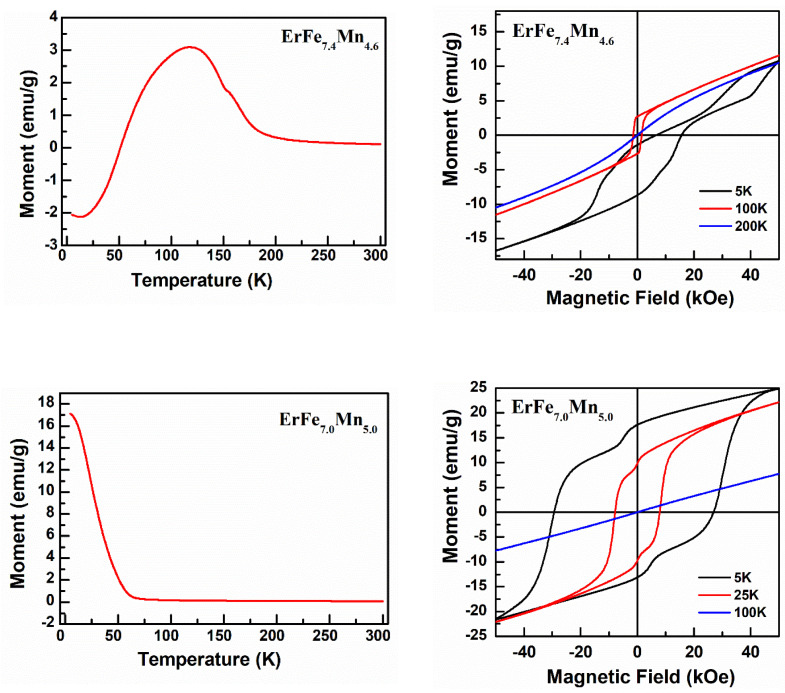
*M*−*T* curve under field cooling condition (H = 1000 Oe) and *M*−*H* curve after 1000 Oe field cooling of ErFe_x_Mn_12−x_ (7.0 ≤ x ≤ 9.0) series alloys.

Figure 4 shows the magnetic phase diagram of the ErFe_x_Mn_12−x_-series (7.0 ≤ x ≤ 9.0) alloys. The samples with x < 7.4 or x > 8.4 were mainly ferromagnetic. The samples with 7.4 ≤ x ≤ 8.2 were ferromagnetic and antiferromagnetic, and only the samples in this range of composition showed antiferromagnetic orders between different transition metal lattices. YFe_x_Mn_12−x_-series samples showed a clear exchange bias effect in the region where ferromagnetic interaction and antiferromagnetic interaction compete most intensely [23]. ErFe_x_Mn_12−x_-series samples may therefore similarly display exchange bias effects for 7.4 ≤ x ≤ 8.2. The FC *M−H* loops of some samples were measured, and the results are shown in Figure 5. The FC *M−H* loops of ErFe_8.2_Mn_3.8_, ErFe_7.8_Mn_4.2_ and ErFe_7.4_Mn_4.6_ samples all clearly had lateral shifts. The x = 7.4 and x = 7.8 samples had high coercivity, and the *M−H* loops were not completely closed when the applied field was 5T. The *M−H* loops were asymmetric, and lateral and vertical shifts occurred simultaneously. This indicates that the samples had very strong magnetocrystalline anisotropy at low temperatures, and that the antiferromagnetic interaction between the rare earth lattice and the transition metal lattice was the source of the anisotropy. When combined with the YFe_x_Mn_12−x_-series experimental results, we see that the exchange bias effect can be controlled by doping different rare earth elements in addition to altering the ratios of Fe and Mn.

**Figure 6:**
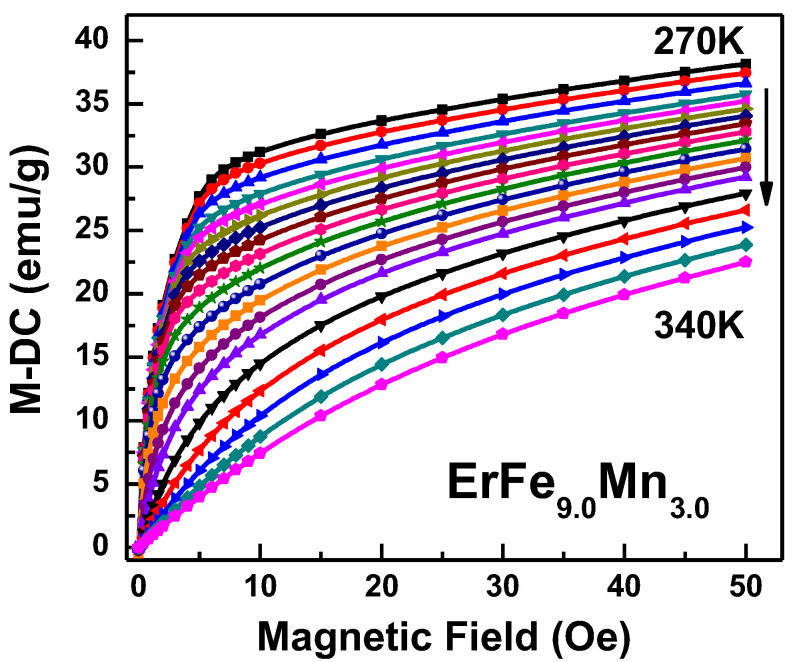
Isothermal magnetization curve of ErFe_9.0_Mn_3.0_.

**Figure 7:**
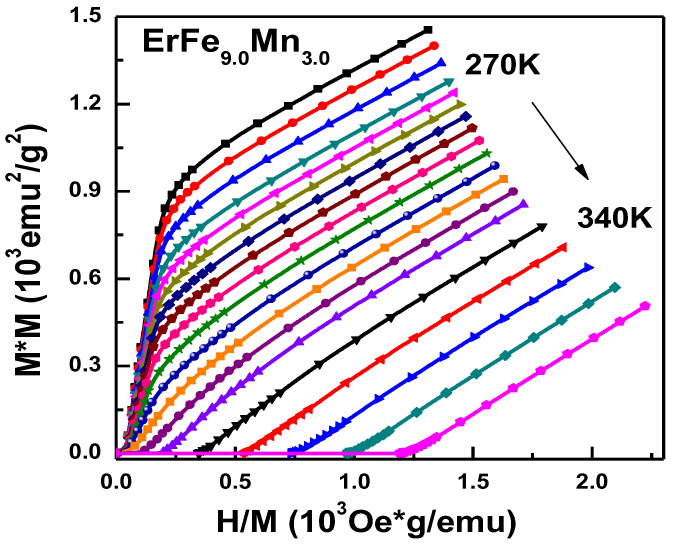
Arrott curve of ErFe_9.0_Mn_3.0_.

The ErFe_9.0_Mn_3.0_ compound had a Curie temperature of 310 K, and which is near the room temperature. The reverse magnetic moment of Er atom is decrease drastically as temperature increasing, so the samples may have had a considerable magnetocaloric effect near the Curie temperature. The isothermal magnetization curves in the temperature range 270–340 K were created, and are shown in Figure 6. The figure shows that as the temperature increased, magnetization intensity gradually decreased, and ferromagnetism was gradually transformed into paramagnetism. The isothermal magnetization curves were transformed to obtain the Arrott plot, as shown in Figure 7, in order to determine the type of phase transition occurring. There was no S-shaped curve in the Arrott plot, and no negative curve slope was observed, so the phase transition of the materials was also a second-order phase transition.

**Figure 8:**
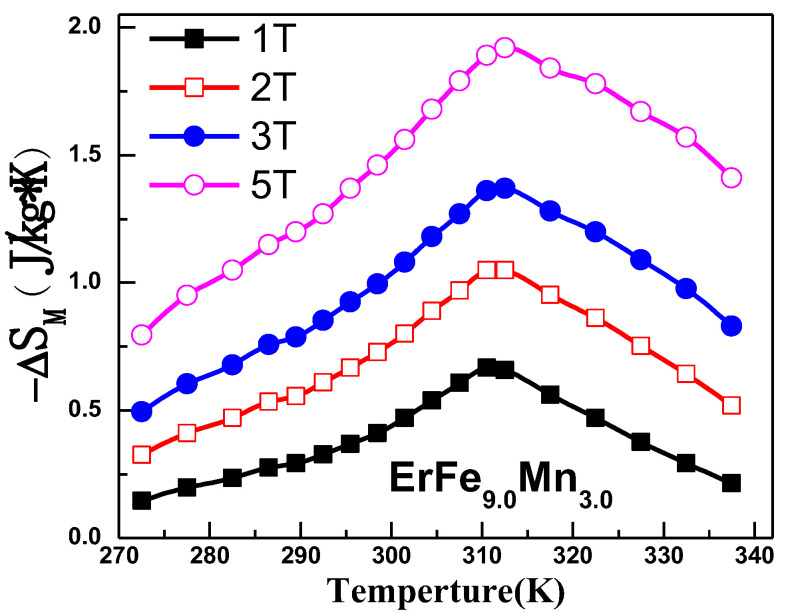
Isothermal magnetic entropy change with temperature for ErFe_9.0_Mn_3.0_.

The Maxwell relation was used to calculate the isothermal magnetic entropy change in the samples from the isothermal magnetization curves at different temperatures, as shown in Figure 8. The calculated maximum value of the magnetic entropy changes when an applied field change of 50 kOe reaches 1.92 J/kg/K. The peak of −Δ*S_M_* at 312.5 K corresponds to the ferromagnetic to paramagnetic phase transition, because the magnetization changes drastically near the Curie temperature. Although the maximum −Δ*S_M_* of ErFe_9.0_Mn_3.0_ is not as large as that of some other magnetic refrigerant materials [28], the *|*Δ*S_M_|* vs. *T* curve of ErFe_9.0_Mn_3.0_ is significantly broader compared with other materials, which is favorable for active magnetic refrigeration. Additionally, the magnetocaloric effect was caused by the second-order phase transition near the Curie temperature, and the thermal hysteresis and magnetic hysteresis during phase transition were both very small, which has benefits in the practical application of the material.

## 4. Conclusions

ThMn_12_-type single phase samples with different Fe/Mn ratios were prepared by arc melting and heat treatment, and the magnetic phase diagrams of ErFe_x_Mn_12−x_-series (7.0 ≤ x ≤ 9.0) samples were obtained by magnetic measurement. At low temperatures, samples with x < 7.4 and x > 8.4 exhibited ferromagnetism, and ferromagnetism and antiferromagnetism coexisted in samples with 7.4 ≤ x ≤ 8.2, with an FC exchange bias effect. The magnetic interaction between transition metal lattices in ThMn_12_-type structural materials can be changed by substituting non-magnetic Y atoms with rare earth Er atoms with magnetic moments. In this study, Y atoms were completely replaced; in the following study, we will partially replace them to finely modulate the exchange bias effect and the magnetocaloric effect of the materials.

## Figures and Tables

**Table 1:** Information on crystal structure parameters of ErFe_x_Mn_12−x_ (7.0 ≤ x ≤ 9.0) series alloys.

ErFe_x_Mn_12−x_	*a*(Å)	*c*(Å)	occ, Fe, 8i	occ, Fe, 8j	occ, Fe, 8f	*n*, Fe	*n*, Mn	*Rwp*
ErFe_9.0_Mn_3.0_	8.45469(11)	4.75397(7)	0.476(0)	0.836(4)	0.928(8)	8.96	3.04	5.11
ErFe_8.6_Mn_3.4_	8.45777(24)	4.75346(16)	0.412(4)	0.788(12)	0.892(16)	8.368	3.632	4.54
ErFe_8.2_Mn_3.8_	8.46289(11)	4.75501(7)	0.344(0)	0.792(4)	0.908(8)	8.176	3.824	4.73
ErFe_7.8_Mn_4.2_	8.46758(16)	4.75572(11)	0.300(0)	0.704(8)	0.832(8)	7.344	4.656	3.88
ErFe_7.4_Mn_4.6_	8.47191(24)	4.75547(16)	0.284(0)	0.640(8)	0.764(8)	6.752	5.248	3.29
ErFe_7.0_Mn_5.0_	8.47767(19)	4.75605(13)	0.232(0)	0.636(4)	0.796(8)	6.656	5.344	7.28

**Table 2:** Magnetic ordering temperature, exchange bias field and coercive force field of ErFe_x_Mn_12−x_ (7.0 ≤ x ≤ 9.0) series alloys.

ErFe_x_Mn_12−x_	*T_C_* (K)	*T_f_* (K)	*T_N_* (K)	*H_E_* (kOe)	*H_C_* (kOe)
Cooling Field	50 Oe	50 Oe	50 Oe	1000 Oe	1000 Oe
ErFe_9.0_Mn_3.0_	310	306			
ErFe_8.6_Mn_3.4_	250	248		−0.22	1.28
ErFe_8.2_Mn_3.8_	208	203	142	11.73	2.97
ErFe_8.0_Mn_4.0_	178	170	163		
ErFe_7.8_Mn_4.2_	154	160	169	6.615	9.54
ErFe_7.4_Mn_4.6_	128	126	176	11.08	4.52
ErFe_7.2_Mn_4.8_	22	44			
ErFe_7.0_Mn_5.0_	22	36		−1.27	28.11

## Data Availability

The data presented in this study are available in this article.
